# Early Cortical Changes in Gamma Oscillations in Alzheimer’s Disease

**DOI:** 10.3389/fnsys.2016.00083

**Published:** 2016-10-26

**Authors:** Alexandra S. Klein, José R. Donoso, Richard Kempter, Dietmar Schmitz, Prateep Beed

**Affiliations:** ^1^Neuroscience Research Center, Charité UniversityBerlin, Germany; ^2^Department of Biology, Institute for Theoretical Biology, Humboldt UniversityBerlin, Germany; ^3^Bernstein Center for Computational NeuroscienceBerlin, Germany; ^4^Cluster of Excellence “NeuroCure”, Charité UniversityBerlin, Germany; ^5^DZNE - German Center for Neurodegenerative DiseasesBerlin, Germany; ^6^Einstein Foundation BerlinBerlin, Germany; ^7^Berlin Institute of HealthBerlin, Germany

**Keywords:** entorhinal cortex, gamma oscillation, Alzheimers disease, presymptomatic, interneurons

## Abstract

The entorhinal cortices in the temporal lobe of the brain are key structures relaying memory related information between the neocortex and the hippocampus. The medial entorhinal cortex (MEC) routes spatial information, whereas the lateral entorhinal cortex (LEC) routes predominantly olfactory information to the hippocampus. Gamma oscillations are known to coordinate information transfer between brain regions by precisely timing population activity of neuronal ensembles. Here, we studied the organization of *in vitro* gamma oscillations in the MEC and LEC of the transgenic (tg) amyloid precursor protein (APP)-presenilin 1 (PS1) mouse model of Alzheimer’s Disease (AD) at 4–5 months of age. *In vitro* gamma oscillations using the kainate model peaked between 30–50 Hz and therefore we analyzed the oscillatory properties in the 20–60 Hz range. Our results indicate that the LEC shows clear alterations in frequency and power of gamma oscillations at an early stage of AD as compared to the MEC. The gamma-frequency oscillation slows down in the LEC and also the gamma power in dorsal LEC is decreased as early as 4–5 months in the tg APP-PS1 mice. The results of this study suggest that the timing of olfactory inputs from LEC to the hippocampus might be affected at an early stage of AD, resulting in a possible erroneous integration of the information carried by the two input pathways to the hippocampal subfields.

## Introduction

The entorhinal cortex integrates information from many sensory modalities and (sub)cortical areas before projecting to the hippocampus proper. The medial entorhinal cortex (MEC) has been described to process and code spatial information (Fyhn et al., 2004; Hafting et al., 2005) whereas the lateral entorhinal cortex (LEC) processes predominantly olfactory inputs (Igarashi et al., 2014). Recent work has elucidated the projection pathways from the entorhinal cortices to the hippocampal subfields at a higher anatomical and functional resolution (Zhang et al., 2013; Kitamura et al., 2014, 2015; Ray et al., 2014). Thus under physiological conditions both the MEC and LEC are critical processing stations of information that is important for memory processes and pathways in the temporal lobe (Van Cauter et al., 2013).

Network activity in the gamma frequency (30–100Hz; for review see Singer and Gray, 1995; Jefferys et al., 1996; Farmer, 1998) plays an important role in information transfer across connected brain regions (Gray et al., 1989; Llinas and Ribary, 1993; Murthy and Fetz, 1996; Steriade et al., 1996; Melzer et al., 2012) and across cortical hemispheres (Shinohara et al., 2013). Such oscillatory activity brings multimodal inputs together in a target region for efficient spatio-temporal integration (Murthy and Fetz, 1996; Steriade et al., 1996). In fact, it has been recently shown that LEC and distal CA1 show a higher coherence in the gamma frequency range during an olfactory task, implying that such oscillatory processes may be important in information transfer and binding (Igarashi et al., 2014). The presence of gamma oscillations has been demonstrated *in vivo* in the hippocampus (Bragin et al., 1995; Traub et al., 1996; Penttonen et al., 1998) and in the entorhinal cortex of the rat (Eeckman and Freeman, 1990; Chrobak and Buzsáki, 1998). *In vivo* gamma frequency can be subdivided into a slow (30–65 Hz) and a fast state (65–100 Hz). In the CA1 region, slow gamma and fast gamma arrive from the CA3 and the entorhinal cortices, respectively (Colgin et al., 2009). Slow gamma state is implicated for memory retrieval whereas the fast gamma state is important during memory encoding stages in the temporal lobe (Bieri et al., 2014).

Alzheimer’s disease (AD) is a well-known neurodegenerative disorder that is characterized by the presence of senile plaques and neurofibrillary tangles (Braak and Braak, 1991; Van Hoesen and Solodkin, 1993). Such plaques and tangles disrupt anatomical (for review see Van Hoesen and Solodkin, 1994) and physiological functions of neurons (for review see Palop et al., 2006; Driver et al., 2007) at the cellular (Dolev et al., 2013), synaptic (Gazit et al., 2016) and circuit levels (Booth et al., 2016b), including disruption of long-range synchrony in the cortex (Busche et al., 2015), hypersynchrony in hippocampal circuits (Verret et al., 2012), and hyperexcitability of cells showing increased firing rates near plaques (Busche et al., 2008). It may be here noted that while some neurons upregulated their firing rate in a local network, others were silenced (Busche et al., 2008). However, the neuronal cell types displaying this differential activity patterns are not yet known. In parallel, it has also been shown that Aβ can traverse synapses, in particular from the entorhinal cortex to the dentate gyrus (Harris et al., 2010).

In post-mortem tissue of patients suffering from neurodegenerative diseases such as AD, the entorhinal cortex is severely affected (Braak and Braak, 1991). In fact, it has been reported that both in humans and mice models of AD, there is extensive neuronal loss and damage in the entorhinal region (Van Hoesen and Solodkin, 1993; Solodkin and Van Hoesen, 1996). Interestingly, the LEC and MEC are affected at an early stage of AD before alterations are seen in the hippocampus proper (Khan et al., 2014). Although the entorhinal cortices offer a great opportunity to test circuit alterations at early stages of AD, very few studies have so far investigated into it.

Processing of olfactory (Wesson et al., 2010; Vasavada et al., 2015) as well as spatial information (Monacelli et al., 2003; Vlček and Laczó, 2014; Kunz et al., 2015) is affected at an early stage of AD. As the LEC conveys mainly olfactory and the MEC conveys mainly spatial information to the hippocampus, gamma oscillations in each of these regions could promote such information transfer between the sources to the target regions. Possible disruption of gamma oscillations at early stages of AD in the entorhinal cortices (Verret et al., 2012; Booth et al., 2016a) would have severe implications on the integration of such inputs in the downstream target areas such as the dentate gyrus, CA3 and CA1 in the hippocampus. Robust gamma oscillations can be induced in slices (Whittington et al., 1995, 1997; Funahashi and Stewart, 1998; Bartos et al., 2007) of the entorhinal cortex using kainate (Cunningham et al., 2003). In this *in vitro* model, gamma oscillatory activity in the 20–100 Hz range was observed in the MEC with peak activity around 40–50 Hz (Cunningham et al., 2003). In our study, using kainate, we investigated the organization of gamma oscillations in both LEC and MEC in a 4–5 months-old transgenic (tg) amyloid precursor protein (APP)-presenilin 1 (PS1) tg mouse model for AD. For the first time we report of robust gamma oscillations in the LEC besides the well documented induction of gamma oscillations in the MEC using this method. Further, we quantified the changes in density of the three main populations of cortical interneurons. Interneurons have been shown to play a key role in orchestrating gamma oscillations (Sohal et al., 2009) and are reported to degenerate in AD (Mikkonen et al., 1999; Baglietto-Vargas et al., 2010). Decrease in the number of interneuronal subtypes could be a causal correlate for the observed changes in gamma oscillations (Verret et al., 2012) in AD pathophysiology.

## Materials and Methods

### Animals

All experimental procedures were performed in accordance with German guidelines on animal welfare under the supervision of local ethics committees (animal license number T100/03). The tg AD model used for this project is the tg APP-PS1 mouse generated by Radde et al. (2006). It coexpresses KM670/671NL mutated APP and L166P mutated PS1 under the control of a neuron-specific Thy1 promoter element. Non-tg littermates of the same age and the same genetic background (C57BL/6) were used as wild type (wt) controls.

For electrophysiological experiments, seven wt and 10 tg (APP-PS1/tg) animals between 4–5 months of age were recorded as in the tg APP-PS1 mouse model that we used, the disease progression is comparable between the 4–5 months of age. For consecutive plaque visualization, three of the tg APP-PS1 and two of the wt mice were systemically injected with 4,4′-[(2-methoxy-1,4-phenylene)di-(1*E*)-2,1-ethenediyl]bisphenol (Methoxy-X04, Klunk et al., 2002). A single dose of 10 μl per g bodyweight of amyloid staining solution (4% vol of 10 mg/ml methoxy-X04 in DMSO, and 7.7% vol CremophorEL (Sigma-Aldrich) in 88.3% vol PBS) was administered intraperitoneally 24 h before preparation of acute brain slices, thus ensuring a homogenous distribution of the compound in the whole brain.

### Preparation of Acute Brain Slices

Horizontal slices of the entorhinal cortex were prepared from tg APP-PS1 mice and age matched wt littermates. Animals were anesthetized and decapitated. The brains were quickly removed into ice-cold sucrose containing artificial cerebral spinal fluid (S-ACSF, containing (mM) 87 NaCl, 26 NaHCO_3_, 10 glucose, 2.5 KCl, 1.25 NaH_2_PO_4_, 3 MgCl_2_, 0.5 CaCl_2_, and 50 sucrose, pH 7.4). Tissue blocks were mounted on a vibratome (VT 1200; Leica Microsystems) with the dorsal side down, and around six slices per animal were cut at 400 μm thickness from the ventral side of the brain. They were stored at 35°C in an interface chamber that consisted of separate storage and recording chambers. Slices from dorsal (slice number 4–6) and ventral levels (slice number 1–3) were ordered accordingly across all experimental days. The interface chamber was perfused with ACSF (containing (in mM) 119 NaCl, 26 NaHCO_3_, 10 glucose, 2.5 KCl, 1 NaH_2_PO_4_, 2.5 CaCl_2_ and 1.3 MgCl_2_) with a perfusion rate of 2.5–3.0 ml/min. Slices were incubated for 60 min before recordings started. All ACSF solutions were equilibrated with carbogen (95% O_2_ and 5% CO_2_).

### Electrophysiological Recordings and Analysis

Recording electrodes with impedance of 2–3 MOhm were pulled from borosilicate glass capillaries (Harvard Apparatus, Kent, UK; 1.5 mm OD) using a micropipette electrode puller (DMZ Universal Puller) and filled with ACSF. The impedance of the electrode did not affect the power of the gamma oscillations. Data were amplified and digitized at 5 kHz using a BNC-2090 adapter chassis (National Instruments, Austin, TX, USA). Voltage was low-pass filtered at 1.7 kHz and recorded in IGOR Pro (WaveMetrics Inc., OR, USA). Brain slices containing MEC and LEC along with the rest of the hippocampal formation were transferred to the recording chamber, the entorhinal cortex was visually identified using a binocular microscope equipped with a 3× objective, and the extracellular recording electrodes were placed in Layer III and close to the border of Layer II of either LEC or MEC, respectively. Baseline activity was recorded for around 5 min before gamma oscillations were evoked by bath application of 400 nM kainate for at least 50–60 min. After recordings were finished, all slices were fixed in 4% paraformaldehyde (PFA) at 4°C for 48 h and afterwards stored in 0.1 M phosphate buffered saline (PBS).

To analyze the frequency content of the signal, we generated spectrograms by convolving the signal with a bank of Gabor wavelets covering the band 5–100 Hz in steps of 2 Hz. The time course of the power and the power spectra were calculated by integrating the spectrogram across frequencies for every time step (sweep) and by averaging the spectrogram across time (average of last 20 sweeps, each sweep was 10 s), respectively. The gamma power was obtained by integrating the power spectral density within the 20–60 Hz range. We have also analyzed the spectral power in the whole gamma band range of 20–100 Hz (data not shown). Gamma power in the 60–100 Hz range reflects the tail of the power spectrum rather than the fast gamma that is observed in *in vivo* recordings. We believe that we did not evoke any fast gamma using kainate. As the peak frequency in the kainate induced *in vitro* gamma oscillations ranged between 30–50 Hz, we have used used gamma power in the 20–60 Hz range unless otherwise mentioned.

### Analysis of Aβ Plaque Density

For visualization of Aβ plaques, three tg animals and two wt animals between 4–5 months of age were treated with Methoxy-X04 24 h before preparation of acute brain slices. After electrophysiological recordings were finished, brain slices were fixed in 4% PFA and mounted onto glass slides with Roti^®^-Mount FluorCare (Roth GmbH and Co KG). Images from 24 slices were acquired using 10× *z*-plane stacks (consisting of 18 images at 5 μm step size) containing MEC and LEC; additionally, a bright-field image was captured for identification of the layers. Aβ plaque load was additionally analyzed in perfused brain tissue from two tg animals at 3 months of age (28 slices). These images were acquired using 10× *z-plane* stacks (consisting of 5 images at 5 μm step size) containing both the MEC and LEC.

Laser intensity and gain were adjusted for every image to maximize the visibility of the plaques and a maximum-intensity *z*-projection of each image was generated using the cellSense software. The regions of interest were manually outlined, and the number of plaques in layers II and III in MEC and LEC was analyzed using the “analyze particle”-plugin of ImageJ. Plaque density was determined for every section as number of plaques normalized to an area of 100 μm^2^ × 100 μm^2^.

### Immunohistochemistry

Early changes in the density of cortical interneurons were analyzed using immunohistochemical stainings. Two tg APP-PS1 and two wt littermate mice between 4–5 months of age were used.

Mice were anesthetized (0.5% ketamine, i.p.) and transcardially perfused with 4% PFA, and brains were postfixed overnight in 4% PFA. Each hemisphere was cut on a vibratome (Leica VT1000S, Leica Biosystems, Nussloch, Germany) into 50 μm thick horizontal slices and transferred to a 24-well plate containing 0.1 M PBS. Non-specific staining was blocked by preincubating the sections in 5% normal goat serum (NGS) and 1% Triton-X-100 in 0.1 M PBS for 1 h. Slices were incubated for 48–72 h at 4°C in a combination of either rabbit anti-calretinin (CR, 1:4000, polyclonal, Swant, 1:2000) plus mouse anti-reelin (1:1000, monoclonal, Millipore) or mouse anti-parvalbumin (PV, monoclonal, 1:5000, Swant) plus rabbit anti-somatostatin (SOM, 1:1000, polyclonal, Bachem/Peninsula Laboratories LLC.,San Carlos, CA, USA). To visualize immunoreactivity, the slices were incubated for 2 h in a combination of anti-rabbit Alexa488 (1:500, Invitrogen, Carlsbad, CA, USA) and anti-mouse Alexa555 (1:500, Invitrogen, Carlsbad, CA, USA) in 0.5% Triton-X-100 in 0.1 M PBS on a shaker at room temperature. After rinsing with 0.1 M PBS, slices were mounted onto glass slides with Roti^®^-Mount FluorCare (Roth GmbH & Co KG).

### Imaging and Image Analysis

Cell densities were visualized using digital images that were acquired with an Olympus BX-61 epifluorescence microscope using the Olympus cellSense digital software (Olympus America Inc., Center valley, PA, USA). The microscope was equipped with a mercury lamp; excitation filters for UV (360–370 nm), blue (470–495 nm) and green (540–550 nm); a CCD camera for detection of emitted blue (420–460 nm), green (510–550 nm) or red (575–625 nm) light; a 2.5×, a 10× and a 20× dry immersion objective. Images were analyzed using the cellSense and the ImageJ software (NIH).

PV, SOM and CR positive interneurons were quantitatively analyzed in MEC and LEC. We analyzed 22–25 wt and 18–22 tg APP-PS1 sections in total. Images of layer II/III were captured with a 20× dry immersion objective (1.2 numerical aperture) and at a resolution of 1392 × 1040 pixels. *Z*-plane stacks consisting of five images were collected with a step size of 5 μm throughout the section depth. Laser intensity and gain were adjusted for each image separately to maximize the visibility of immunoreactive cells. A maximum intensity *z-projection* was generated, and the area of interest (layer II/III) was manually outlined and measured, brightness and contrast were optimized, and the color images were converted to binary images using the “auto threshold” function. Cells were automatically counted using the “analyze particle”-plugin. The number of cells was normalized to an area of 100 μm^2^ × 100 μm^2^, and interneuron density was then defined as (number of interneurons/10,000 μm^2^) for every section.

### Statistical Analysis

Data are expressed as mean ± SEM. All statistical testing assumed a non-parametric distribution and a *Mann-Whitney* test was used; *n.s.*: non significant; **p < 0.01, *p < 0.05.

## Results

The entorhinal cortices (medial and lateral subdivisions) in the temporal lobe of the brain are key structures relaying memory related information between the neocortex and the hippocampus. The MEC routes spatial information, whereas the LEC routes predominantly olfactory information to the hippocampus. Gamma oscillations are known to coordinate information transfer between brain regions by precisely timing population activity of neuronal ensembles. Here we quantified *in vitro* gamma oscillations in the MEC and LEC of the tg APP-PS1 mouse model of AD.

### Medial and Lateral Entorhinal Cortices Oscillate at Different Gamma Frequencies

**Figure 1:**
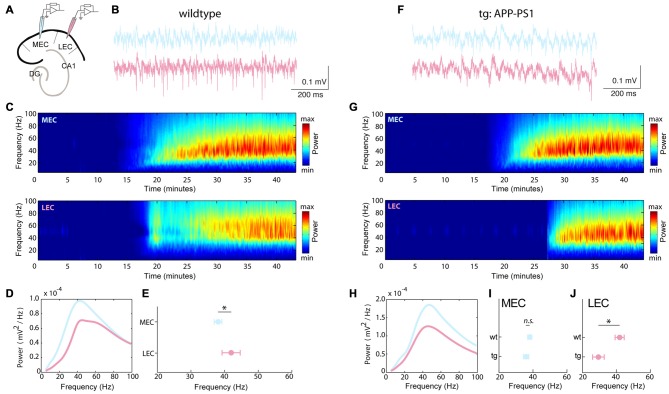
**Frequency of *in vitro* gamma oscillations slow down in the lateral but not the medial entorhinal cortex (MEC) in the transgenic (tg) amyloid precursor protein (APP)-presenilin 1 (PS1) mice as early as 4–5 months.** Gamma oscillations were evoked using kainate and stable recordings were established in both the MEC and lateral entorhinal cortex (LEC): **(A–E)** wild type (wt) and **(F–H)** tg APP-PS1. **(A)** Recording configurations for simultaneous gamma oscillation recordings in the MEC and LEC. **(B)** Local field potential recordings from MEC (blue) and LEC (pink) showing gamma oscillatory activity. **(C)** Spectrograms showing the frequency content of the signal (from the same experiment as in **B**). **(D)** Power spectra of the same experiment as in **(B)**. **(E)** Peak frequency of gamma oscillations in the LEC and MEC (LEC: 42.00 ± 2.76 Hz; MEC: 38.14 ± 1.54 Hz, *n* = 21 recordings from seven wt animals; *p* = 0.0168). **(F)** Local field potential recordings from MEC (blue) and LEC (pink) showing gamma oscillatory activity in the tg APP-PS1 mice. **(G)** Spectrograms showing the frequency content of the signal (from the same experiment as in **F**). **(H)** Power spectra of the same experiment as in **(F)**. **(I,J)** Peak frequency of gamma oscillations for **(I)** MEC wt and tg APP-PS1 animals show no significant differences (MEC wt: 38.14 ± 1.54 Hz; MEC tg: 36.00 ± 1.54 Hz, *n* = 21 MEC recordings from seven wt animals, 37 MEC recordings from 10 tg animals; *p* = 0.168). **(J)** LEC wt and tg APP-PS1 peak frequencies are significantly different (LEC wt: 42.00 ± 2.76 Hz; LEC tg: 29.54 ± 3.43 Hz, *n* = 21 LEC recordings from seven wt animals, 37 LEC recordings from 10 tg animals; *p* = 0.0107). *n.s.*–non significant, *p < 0.05, **p < 0.01.

The MEC is known to generate robust gamma oscillations in both *in vitro* and *in vivo* studies (Cunningham et al., 2003; Beed et al., 2013). Recently an *in vivo* study showed that the LEC transfers odor related information to the hippocampal CA1 area by synchronizing the two areas at the gamma frequency range (Igarashi et al., 2014). However to date there is no *in vitro* evidence of gamma oscillations in the LEC. Using an *in vitro* kainate model of gamma oscillations, we simultaneously measured robust and reliable gamma oscillations in both the MEC and LEC (success rate of 78.37% in wt recordings, i.e., 29 out of 37 slices; Figure 1). The LEC gamma was on average faster than the MEC gamma (LEC: 42.00 ± 2.76 Hz; MEC: 38.14 ± 1.54 Hz, *n* = 21 recordings; *p* = 0.0168; Figure 1E).

### Frequency of Gamma Oscillations Slow Down in the Lateral Entorhinal Cortex Early-on in tg APP-PS1 Mice

Early alterations in network activity have been observed in the hippocampus in AD (Palop et al., 2006). The entorhinal cortex is particularly susceptible to early onset of AD. As we were interested in characterizing early alterations in network activity, we recorded gamma oscillations in 4–5 month-old tg APP-PS1 tg mice (Figures 1F–H). In the tg recordings, we could evoke gamma oscillations with a comparable success rate (in 72.88% of the recordings, i.e., 43 out of 59 slices) to wt littermates. We observed that the frequency of gamma in the tg LEC significantly slowed down as compared to the wt (LEC wt: 42.00 ± 2.76 Hz; LEC tg: 29.54 ± 3.43 Hz, *n* = 21 LEC wt, 37 LEC tg recordings; *p* = 0.0107; Figure 1J). However, the frequency in the MEC was unaltered between the two genotypes (MEC wt: 38.14 ± 1.54 Hz; MEC tg: 36.00 ± 1.54 Hz, *n* = 21 MEC wt, 37 MEC tg recordings; *p* = 0.168; Figure 1I).

### Organization and Development of Gamma Power in the Medial and Lateral Entorhinal Cortices

**Figure 2:**
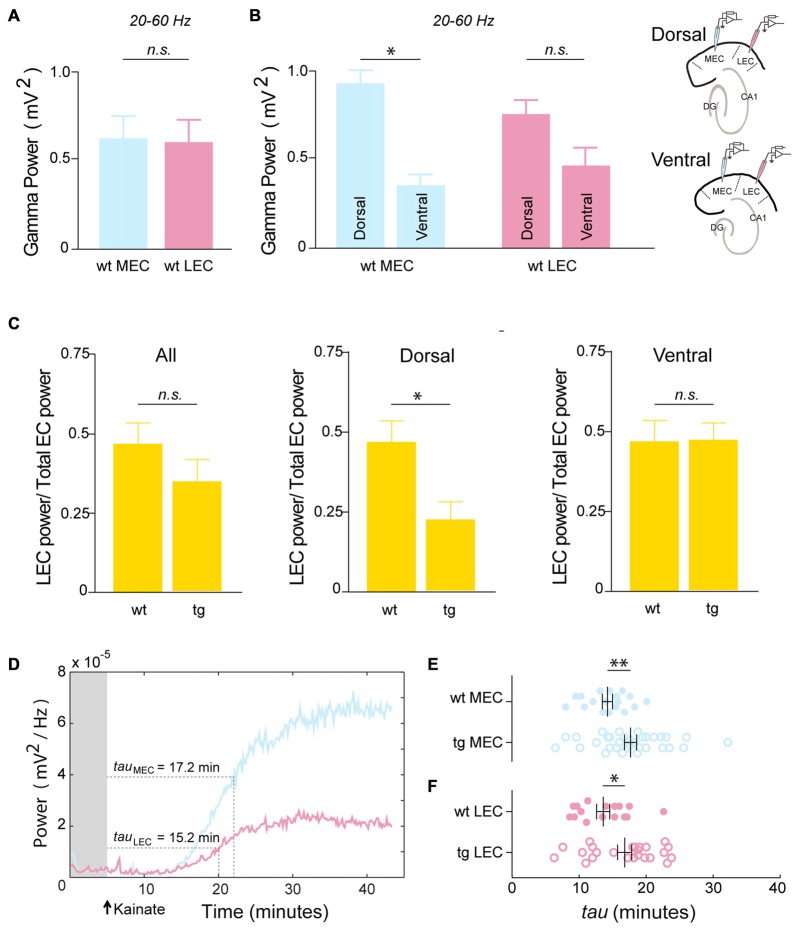
**Dorsal LEC show early alterations in gamma power.** Organization and development of gamma power in the medial and LEC: **(A)** In the wt mice the overall gamma power is not significantly different between MEC and LEC (MEC Power: 0.63 ± 0.13 mV^2^, LEC Power: 0.60 ± 0.13 mV^2^, *n* = 26 recordings; *p* = 0.3204). **(B)** Wt MEC shows dorso-ventral differences in gamma power (MEC Dorsal Power: 0.93 ± 0.23 mV^2^, MEC Ventral Power: 0.36 ± 0.08 mV^2^, *n* = 12 Dorsal, 14 Ventral recordings; *p* = 0.0110). A similar but shallower gradient is seen in the wt LEC (LEC Dorsal Power: 0.76 ± 0.18 mV^2^, LEC Ventral Power: 0.47 ± 0.17 mV^2^, *n* = 12 Dorsal, 14 Ventral recordings; *p* = 0.0584). Recording configurations for the dorso-ventral recordings are shown on the panel on the right. The power of gamma oscillations in the tg APP-PS1 was compared to the wt by quantifying the proportional contribution of the LEC gamma power to the total EC gamma power **(C)** Left panel—The contribution of LEC to the total EC power (LEC power/(LEC power + MEC power)) show no significant differences in the pooled data (LEC power/Total EC power wt: 0.44 ± 0.04, tg: 0.37 ± 0.05; *n* = 26 wt, 40 tg recordings; *p* = 0.1424). Middle panel—Gamma power in the dorsal LEC is severely and significantly reduced in the tg APP-PS1 as compared to the wt (LEC power/Total EC power wt: 0.43 ± 0.06, tg: 0.23 ± 0.07; *n* = 12 Dorsal wt, 18 Dorsal tg recordings; *p* = 0.0181). Right panel—Contribution of LEC power to the total EC power remain comparable between the tg APP-PS1 and wt in the ventral circuits (LEC power/Total EC power wt: 0.44 ± 0.07, tg: 0.48 ± 0.06; *n* = 14 Ventral wt, 22 Ventral tg recordings; *p* = 0.3190). **(D)** Time-course of the development of gamma power in the MEC and LEC. Rise-time constants reflect the time needed for the gamma power to reach 63.2% of the respective maxima. **(E,F)** Rise-time constants for **(E)** MEC and **(F)** LEC show a larger variability in the tg APP-PS1 as compared to the wt mice (MEC wt Rise time: 14.26 ± 0.76 min, MEC tg Rise time: 17.70 ± 0.91 min, *n* = 18 MEC wt, 34 MEC tg recordings; *p* = 0.0048. LEC wt Rise time: 13.60 ± 0.98 min, LEC tg Rise time: 16.80 ± 1.06 min, *n* = 16 LEC wt, 24 LEC tg recordings; *p* = 0.0106). *n.s.*-non significant, *p < 0.05, **p < 0.01.

We next analyzed the organization of gamma power in both MEC and LEC. In the 4–5 month-old wt mice, we did not see any significant differences between the MEC and LEC in the population average (MEC Power: 0.63 ± 0.13 mV^2^, LEC Power: 0.60 ± 0.13 mV^2^, *n* = 26 recordings; *p* = 0.3204; Figure 2A). However, as reported earlier by our group in rats (Beed et al., 2013), here we also observed a difference in gamma oscillation along the dorso-ventral axis in the MEC (higher gamma power at the dorsal levels than ventral ones), suggesting that the organization of physiological gamma activity might be similar between rodent species in the MEC. In particular, we found a 3-fold higher power in dorsal MEC than ventral MEC (MEC Dorsal Power: 0.93 ± 0.23 mV^2^, MEC Ventral Power: 0.36 ± 0.08 mV^2^, *n* = 12 Dorsal, 14 Ventral recordings; *p* = 0.0110; Figure 2B). We also checked whether there was such a dorso-ventral difference in gamma activity in the LEC. Though there is a similar trend in the LEC, the difference between the gamma power at the dorsal and ventral LEC is shallower and not significant (LEC Dorsal Power: 0.76 ± 0.18 mV^2^, LEC Ventral Power: 0.47 ± 0.17 mV^2^, *n* = 12 Dorsal, 14 Ventral recordings; *p* = 0.0584; Figure 2B).

### Dorsal Lateral Entorhinal Cortex Circuits Show the Earliest Alterations in Gamma Power

Gamma oscillations exhibit an organizational gradient along the dorso-ventral axis in the entorhinal cortex (Beed et al., 2013). It has also been shown that different cortical inputs are routed through the hippocampus via the dorsal and ventral circuits in the entorhinal cortices (both MEC and LEC, Canto et al., 2008). Therefore, we analyzed the gamma power at the dorsal and ventral levels for both MEC and LEC in the tg APP-PS1 mice. In each recording configuration we had one electrode in the MEC and the second one in LEC, either at the dorsal or the ventral level, for normalization of gamma power in each experiment (see Supplementary Figure 1). We quantified the contribution of the LEC gamma power to the whole EC gamma power. We found no significant differences between wt and tg animals in the LEC contribution to the total EC gamma power (LEC power/Total EC power wt: 0.44 ± 0.04, tg: 0.37 ± 0.05; *n* = 26 wt, 40 tg recordings; *p* = 0.1424; Figure 2C left). However, when we measured at the dorsal and ventral levels separately, we observed a significantly lower LEC contribution to the total EC power in dorsal slices in the tg animals when compared to their wt littermates (LEC power/Total EC power wt: 0.43 ± 0.06, tg: 0.23 ± 0.07; *n* = 12 Dorsal wt, 18 Dorsal tg recordings; *p* = 0.0181; Figure 2C middle). In the ventral slices we observed no difference between the two genotypes (LEC power/Total EC power wt: 0.44 ± 0.07, tg: 0.48 ± 0.06; *n* = 14 Ventral wt, 22 Ventral tg recordings; *p* = 0.3190; Figure 2C right).

### Development of Gamma Power is More Variable in tg APP-PS1 Mice

In the kainate model of *in vitro* gamma oscillations, a synchronous inhibitory circuitry has been proposed to mediate gamma activity (Bartos et al., 2007). The development and strength of gamma power has been attributed to the architecture of the circuitry (Cunningham et al., 2003). To investigate the development of gamma power upon kainate application in the entorhinal cortices, we quantified the rise time for the gamma power to reach its peak value (Figure 2D). In a recent article of Tauopathy (Booth et al., 2016a) the authors use the rise time of gamma power as a correlative evidence for the neuronal circuit related impairments. In our study, we observed no significant differences in the rise time constant between the wt MEC and wt LEC (MEC Rise time: 14.26 ± 0.76 min, LEC Rise time: 13.60 ± 0.98 min, *n* = 18 MEC, 16 LEC recordings; *p* = 0.2560; Figures 2E,F top rows).

In comparison to the wt littermates, the tg animals showed a more variable distribution in the gamma power both in the MEC and LEC (Figures 2E,F). On average, in both the MEC and LEC of tg animals, gamma oscillations took significantly longer to develop, as seen by the longer rise time constants (MEC wt Rise time: 14.26 ± 0.76 min, MEC tg Rise time: 17.70 ± 0.91 min, *n* = 18 MEC wt, 34 MEC tg recordings; *p* = 0.0048; Figure 2E; LEC wt Rise time: 13.60 ± 0.98 min, LEC tg Rise time: 16.80 ± 1.06 min, *n* = 16 LEC wt, 24 LEC tg recordings; *p* = 0.0106; Figure 2F). This suggested that the gamma generating circuits in tg animals could be impaired at early stages of AD, as already shown in other mouse models (Verret et al., 2012).

### Plaque Load in the Lateral Entorhinal Cortex Leads the Medial Entorhinal Cortex

**Figure 3:**
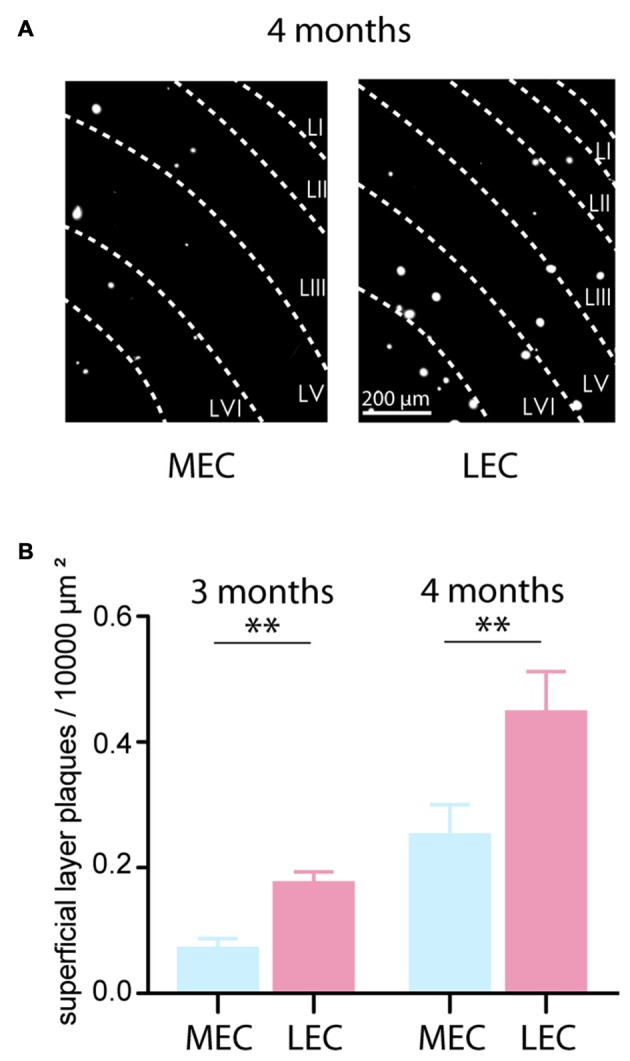
**Plaque load in the LEC leads the MEC. (A)** Aβ plaques were visualized in the MEC and LEC by Methoxy-X04 staining procedure. Plaques in the superficial layers II and III were quantified. The example shows plaque load from 4 months old tg APP-PS1 mice from the dorsal MEC and LEC. **(B)** Left: At 3 months already, the LEC had a significantly greater plaque density in the superficial layers suggesting that LEC rather than the MEC is the starting point of plaque deposition in the entorhinal cortex in the tg APP-PS1 mice (MEC plaque load: 0.07 ± 0.02 plaques per 10,000 μm^2^, LEC plaque load: 0.18 ± 0.02 plaques per 10,000 μm^2^, *n* = 28 slices, *N* = 2 tg animals; *p* < 0.0001). Right: LEC and MEC plaque density at 4 months old tg APP-PS1 mice (MEC plaque load: 0.25 ± 0.05 plaques per 10,000 μm^2^, LEC plaque load: 0.45 ± 0.06 plaques per 10,000 μm^2^, *n* = 24 slices, *N* = 3 tg animals; *p* = 0.0058). *n.s.*–non significant, *p < 0.05, **p < 0.01.

At 4 months, we observed plaque deposition in the entorhinal cortices of the tg APP-PS1 mice (Figure 3A shows examples from the dorsal MEC and LEC). We quantified the plaque load as plaque density per 10,000 μm^2^ in the superficial layers (layers II–III) of both the MEC and LEC. At this early time point, the LEC contributed significantly more to the plaque load in the entorhinal cortex (MEC plaque load: 0.25 ± 0.05 plaques per 10,000 μm^2^, LEC plaque load: 0.45 ± 0.06 plaques per 10,000 μm^2^, *n* = 24 slices, *N* = 3 tg animals; *p* = 0.0058; Figure 3B right). In fact, the LEC leads the MEC in plaque deposition even at an earlier time point. From perfused brains of 3 months old tg APP-PS1, we found a similar trend in plaque deposition between the LEC and MEC (MEC plaque load: 0.07 ± 0.02 plaques per 10,000 μm^2^, LEC plaque load: 0.18 ± 0.02 plaques per 10,000 μm^2^, *n* = 28 slices, *N* = 2 tg animals; *p* < 0.0001; Figure 3B left). As reported by others, we confirmed that the LEC leads the MEC in plaque deposition (Khan et al., 2014).

### Early Changes in Interneuron Numbers in the Entorhinal Cortices

**Figure 4:**
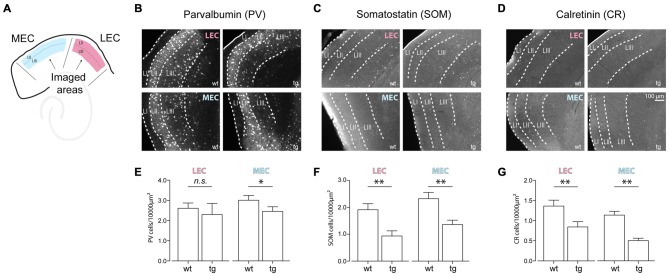
**Early changes in the density of cortical interneurons in the entorhinal cortices in the tg APP-PS1 mice. (A)** Cartoon showing the imaged areas in the MEC and LEC. **(B–D)** Top panel shows dorsal LEC wt and tg and bottom panel shows dorsal MEC wt and tg for **(B)** Parvalbumin (PV; **C**) Somatostatin (SOM) and **(D)** Calretinin (CR) positive interneurons. **(E–G)** Quantification of the density of the three classes of cortical interneurons showed a larger decrease for the **(F)** SOM and **(G)** CR positive (SOM LEC—wt: 1.91 ± 0.22 cells per 10,000 μm^2^ vs. tg: 0.93 ± 0.19 cells per 10,000 μm^2^, *n* = 24 LEC wt, 18 LEC tg slices; *p* = 0.0005; SOM MEC—wt: 2.32 ± 0.23 cells per 10,000 μm^2^ vs. tg: 1.36 ± 0.16 cells per 10,000 μm^2^, *n* = 23 MEC wt, 22 MEC tg slices; *p* = 0.0006. CR LEC—wt: 1.36 ± 0.15 cells per 10,000 μm^2^ vs. tg: 0.84 ± 0.13 cells per 10,000 μm^2^, *n* = 24 LEC wt, 21 LEC tg slices; *p* = 0.0052; CR MEC—wt: 1.14 ± 0.09 cells per 10,000 μm^2^ vs. tg: 0.50 ± 0.06 cells per 10,000 μm^2^, *n* = 25 MEC wt, 19 MEC tg slices; *p* < 0.0001) interneurons as compared to the **(E)** PV (PV LEC—wt: 2.61 ± 0.26 cells per 10,000 μm^2^ vs. tg: 2.30 ± 0.55 cells per 10,000 μm^2^, *n* = 23 LEC wt, 18 LEC tg slices; *p* = 0.0531; PV MEC—wt: 3.01 ± 0.23 cells per 10,000 μm^2^ vs. tg: 2.46 ± 0.23 cells per 10,000 μm^2^, *n* = 22 MEC wt, 18 MEC tg slices; *p* = 0.0500) positive interneurons at 4–5 months of age in the tg APP-PS1 mice. *n.s.*–non significant, *p < 0.05, **p < 0.01.

Inhibition provided onto principal cells, either by soma- or dendrite-targeting interneurons, plays a central role in organizing oscillatory activity, especially in the gamma frequency range (Bartos et al., 2007; Sohal et al., 2009). As reported by others (Mikkonen et al., 1999; Verret et al., 2012), interneuronal dysfunction particularly the PV positive interneurons, results in distorted synchrony in hippocampal circuitry in AD mouse models. We quantified the distribution of the three predominant subpopulations of cortical interneurons—PV, SOM and CR positive cells. We counted the cell density (cells/10,000 μm^2^) of these interneurons in the LEC and MEC at 4–5 months in both wt and tg mice (Figure 4A). In LEC and MEC we found a reduction in the cell density in all the three subpopulations. SOM and CR interneurons showed significant reductions at 4–5 months of age between the tg and littermate wt mice (SOM LEC—wt: 1.91 ± 0.22 cells per 10,000 μm^2^ vs. tg: 0.93 ± 0.19 cells per 10,000 μm^2^, *n* = 24 LEC wt, 18 LEC tg slices; *p* = 0.0005 and SOM MEC—wt: 2.32 ± 0.23 cells per 10,000 μm^2^ vs. tg: 1.36 ± 0.16 cells per 10,000 μm^2^, *n* = 23 MEC wt, 22 MEC tg slices; *p* = 0.0006; Figures 4C,F. CR LEC—wt: 1.36 ± 0.15 cells per 10,000 μm^2^ vs. tg: 0.84 ± 0.13 cells per 10,000 μm^2^, *n* = 24 LEC wt, 21 LEC tg slices; *p* = 0.0052 and CR MEC—wt: 1.14 ± 0.09 cells per 10,000 μm^2^ vs. tg: 0.50 ± 0.06 cells per 10,000 μm^2^, *n* = 25 MEC wt, 19 MEC tg slices; *p* < 0.0001; Figures 4D,G). Surprisingly, unlike the hippocampal CA1 region (Verret et al., 2012), we observed little change in the cell density for the PV interneurons (PV LEC—wt: 2.61 ± 0.26 cells per 10,000 μm^2^ vs. tg: 2.30 ± 0.55 cells per 10,000 μm^2^, *n* = 23 LEC wt, 18 LEC tg slices; *p* = 0.0531 and PV MEC—wt: 3.01 ± 0.23 cells per 10,000 μm^2^ vs. tg: 2.46 ± 0.23 cells per 10,000 μm^2^, *n* = 22 MEC wt, 18 MEC tg slices; *p* = 0.0500; Figures 4B,E).

## Discussion

In our study, we used an *in vitro* model of gamma oscillations to quantify early alterations in cortical gamma oscillatory activity in the medial and lateral subdivisions of the entorhinal cortex.

Recent studies have focused on gaining a deeper understanding of neuronal alterations in the earlier phases of disease progression in AD (Khan et al., 2014; Marcantoni et al., 2014; Kunz et al., 2015). We observed that at 4–5 months of age, the gamma oscillatory activity in the LEC of the tg APP-PS1 mouse shows significant alterations in peak gamma frequency as compared to the age-matched wt littermates. Notably, at the same time point, we found little difference in the gamma oscillatory activity in the MEC between the two genotypes. Further, we observed that the dorsal LEC circuits are particularly susceptible to early alterations in gamma power. Both desynchronized network states (Verret et al., 2012) and disrupted long-range connections (Busche et al., 2015) have been reported in AD. Our study shows that in an *in vitro* model of gamma oscillations, LEC inputs to the hippocampus might be preferentially affected at early stages of AD. It might be of interest to test this hypothesis in a more behaviorally relevant *in vivo* study, for example, investigating the effects of AD pathology on LEC—CA1 synchrony (Igarashi et al., 2014).

Alterations in synaptic transmission and network activity could appear several years (in human) or months (in rodents) before the symptomatic stages. Olfactory activity (Wesson et al., 2010; Vasavada et al., 2015) as well as spatial orientation (Monacelli et al., 2003; Vlček and Laczó, 2014; Kunz et al., 2015) is affected early on in AD mice models, AD patients or those at a higher predisposition risk for AD. Our gamma oscillation data correlates well with other studies showing that dysfunctions in the preclinical disease stages could originate in the LEC and precede the MEC as shown by using high-resolution functional magnetic resonance imaging (Khan et al., 2014). In the LEC, fan cells transmitting the majority of the sensory information to the hippocampus were shown to have altered firing properties in 4-month old Tg2576 mice model (Marcantoni et al., 2014). The LEC mediates predominantly olfactory inputs into the hippocampus proper whereas, the MEC routes more spatial information (Knierim et al., 2013; Igarashi et al., 2014). As gamma oscillations are suggested to be crucial for binding of information across connected brain regions and in long-range synchrony for information transfer (Buzsáki and Wang, 2012), our results indicate that there may be an earlier alteration in the processing of olfactory, rather than spatial, information in the entorhinal cortices in the tg APP-PS1 mice (Vasavada et al., 2015).

Cumulative evidence suggests that LEC circuitry is more susceptible to Alzheimer’s related damage, and precedes degeneration in the MEC in the early stages of the disease (Khan et al., 2014; Marcantoni et al., 2014). We provide additional evidence for this theory by quantifying the plaque load in the LEC and the MEC, and finding that the former substantially preceded the latter. What could be the reason for such differences? One reason could be that the AD-related stresses on metabolic demand or buildup of toxic Aβ affect the LEC and MEC differently. On the other hand, the LEC circuitry itself, i.e., connectivity profile between inhibitory and excitatory neurons could be more sensitive than the MEC. It is still a matter of debate if plaque load has a causal effect on the physiological changes in the various AD mice models. While Hsia et al. (1999) reported that circuit dysfunction is independent of plaque deposition, Grienberger et al. (2012) observed a parallel development in plaque load and physiological alterations. In our study, we report the density of plaques to show that there is a correlative increase of plaque load and the disruption of gamma oscillations in the LEC than in the MEC.

Neuronal loss has been reported in AD in tissue surrounding plaques (Braak and Braak, 1991). Baglietto-Vargas et al. (2010) and Verret et al. (2012) reported that interneurons rather than excitatory neurons are affected earlier (around 3–4 months) in AD mice models. In addition, interneurons play a critical role in orchestrating gamma oscillations (Sohal et al., 2009) therefore we compared the densities of the three main subpopulations of cortical interneurons from the superficial layers of LEC and MEC. Quantification of interneuron cell densities at the same developmental time point where we observed the changes in gamma oscillatory activity revealed a stronger and more significant decrease in the SOM and CR subpopulations in contrast to the PV subpopulation. We included the quantification of interneurons from the superficial layers (layer II and III) as we performed our gamma oscillation measurements also in the same layers It is well known that PV interneurons influence the power of gamma oscillations (Sohal et al., 2009), however the roles of SOM and CR in gamma oscillations are less clear. Further studies in this direction, such as targeted single-cell characterization during oscillatory activity, might reveal a more specific role of the SOM and CR interneurons in entorhinal gamma oscillations. These findings open up the possibility that perhaps different circuits and interneuronal populations modulate the frequency and power of gamma oscillations in the cortex.

In conclusion, the results of this study show that gamma oscillations are altered as early as 4–5 months of age in the tg APP-PS1 mouse model for AD. LEC precedes the MEC in the onset of AD related gamma oscillatory alterations characterized by both a slowing down of gamma frequency and a reduction in the gamma power. Our findings of correlated changes in gamma oscillatory activity and the vulnerability of different interneuronal populations might pave the way for designing rescue strategies for cortical oscillations in the early stages of AD, such as cell-targeted pharmacological treatment.

Little is known about the integration of olfactory and spatial information in the hippocampus. Do rodents use olfactory cues in preference to visual cues to navigate through space? What is the role of the hippocampal formation in olfactory memory? Is this interrupted early on in AD? Our results therefore suggest the need to better understand the integration of inputs from the LEC and MEC in the various hippocampal subfields, both in normal physiology and especially in pathophysiology of AD.

## Author Contributions

AK and PB performed experiments, JRD and RK analyzed the data, AK, DS and PB designed the experiments, RK, DS and PB wrote the manuscript.

## Conflict of Interest Statement

The authors declare that the research was conducted in the absence of any commercial or financial relationships that could be construed as a potential conflict of interest.
