# Supplementary material for: Early Cortical Changes in Gamma Oscillations in Alzheimer’s Disease
Source: Front Syst Neurosci. 2016 Oct 26;10:83. doi: 10.3389/fnsys.2016.00083 (PMC5080538; doi:10.3389/fnsys.2016.00083)
Supplement: Supplementary file 1 [file DataSheet_1.pdf]

Supplementary Figure 1:

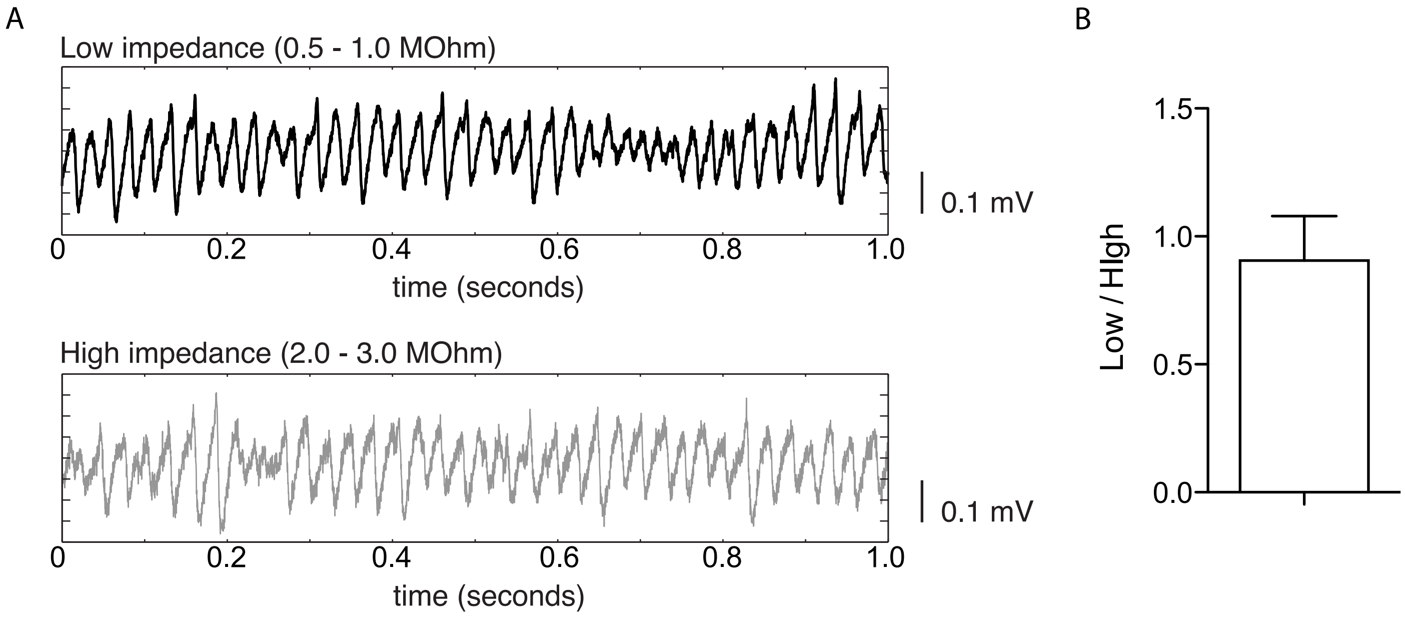

A) Electrode impedance: kainate induced gamma oscillation recordings were performed with low- and high-impedance electrodes and gamma power was analyzed. B) Ratio of gamma power measured by low-impedance / high-impedance electrodes was 0.9060 +/- 0.1732, with a $p = 0.41$ when compared to a ratio of low-impedance / high-impedance of 1.0. There was no statistically significant difference in the measured gamma power between the electrodes of different impedances (low-impedance: 0.5-1.0 MOhm and high-impedance: 2.0-3.0 MOhm).
